# Supplementary material for: Genome-wide antibiotic-CRISPRi profiling identifies LiaR activation as a strategy to resensitize fluoroquinolone-resistant Streptococcus pneumoniae
Source: Nat Commun. 2025 Jul 14;16:6491. doi: 10.1038/s41467-025-61814-x (PMC12260033; doi:10.1038/s41467-025-61814-x)
Supplement: Supplementary file 1 — Supplementary information [file 41467_2025_61814_MOESM1_ESM.pdf]

Genome-wide antibiotic-CRISPRi profiling identifies LiaR activation as a strategy to resensitize fluoroquinolone-resistant *Streptococcus pneumoniae*

Supplementary Information

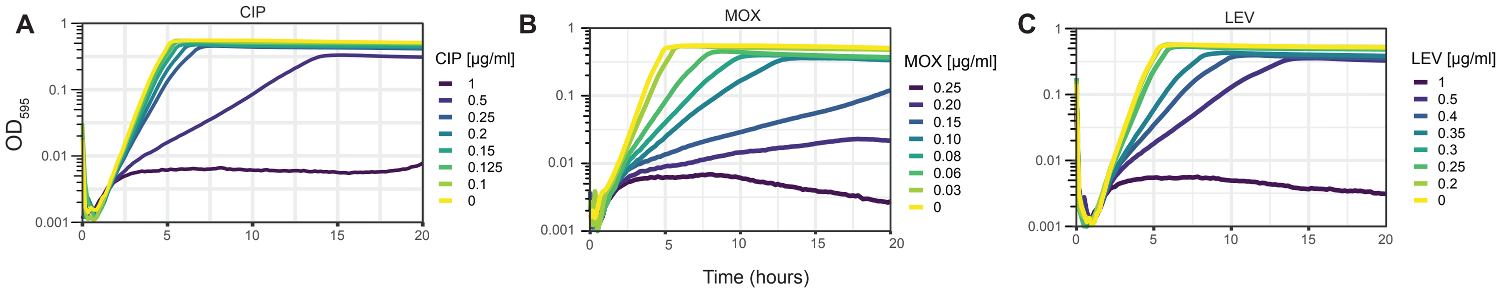

Supplementary Fig 1. Growth curves of the *S. pneumoniae* CRISPRi library treated with a range of antibiotic concentrations. The *S. pneumoniae* CRISPRi library was treated with a panel of fluoroquinolones at varying concentrations to determine the sub-lethal dose. Growth curves are shown here for **A)** ciprofloxacin (CIP) **B)** moxifloxacin (MOX) and **C)** levofloxacin (LEV). Sub-lethal concentrations used for the screens were 0.5, 0.06, and 0.35 µg/ml respectively. Growth curve data represents the mean ± SEM of three biological replicates (n= 3). Source data are provided as a Source Data file.

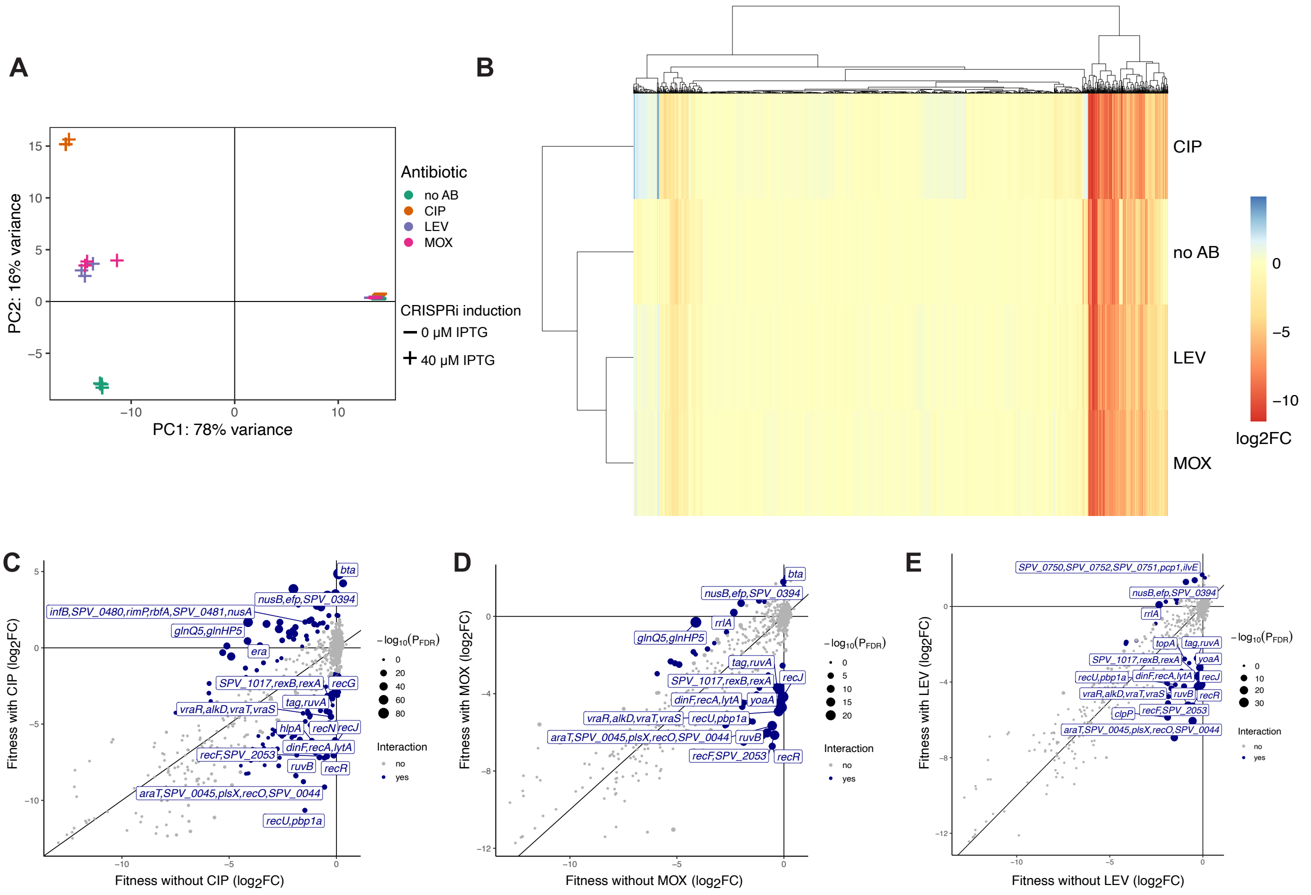

Supplementary Fig 2. CRISPRi-seq screen with *S. pneumoniae* library treated with fluoroquinolones. (A) Principal component analysis served as a quality control measure and indicated distinct clusters between induced and uninduced samples, as well as between antibiotic treated and untreated samples (no AB). Screens were conducted in biological triplicate (n=3) and each point represents a single replicate. **(B)** Heatmap depicting scaled fitness scores of all sgRNA targets. This provides a broad view of significant differential gene fitness with no antibiotic treatment and with antibiotic treatment. A negative log2 fold change (log2FC) score (red) indicates a fitness loss upon gene knockdown and a positive score (blue) indicates a fitness gain compared to no antibiotic. **C-E)** Scatter plots showing genes with significant differential fitness effects (|Δlog2FC| > 1, P_{adj} < 0.05) for each fluoroquinolone screen for ciprofloxacin (CIP), moxifloxacin (MOX) and levofloxacin (LEV). Screens were conducted *in vitro* in C+Y medium. Fitness scores are measured by the log2FC of sgRNA counts between induced and uninduced samples. Points in blue indicate a significant interaction between induction and antibiotic treatment and points in grey indicates a non-significant interaction. Point size indicates the -log10 transformed P_{adj} value. Source data are provided as a Source Data file.

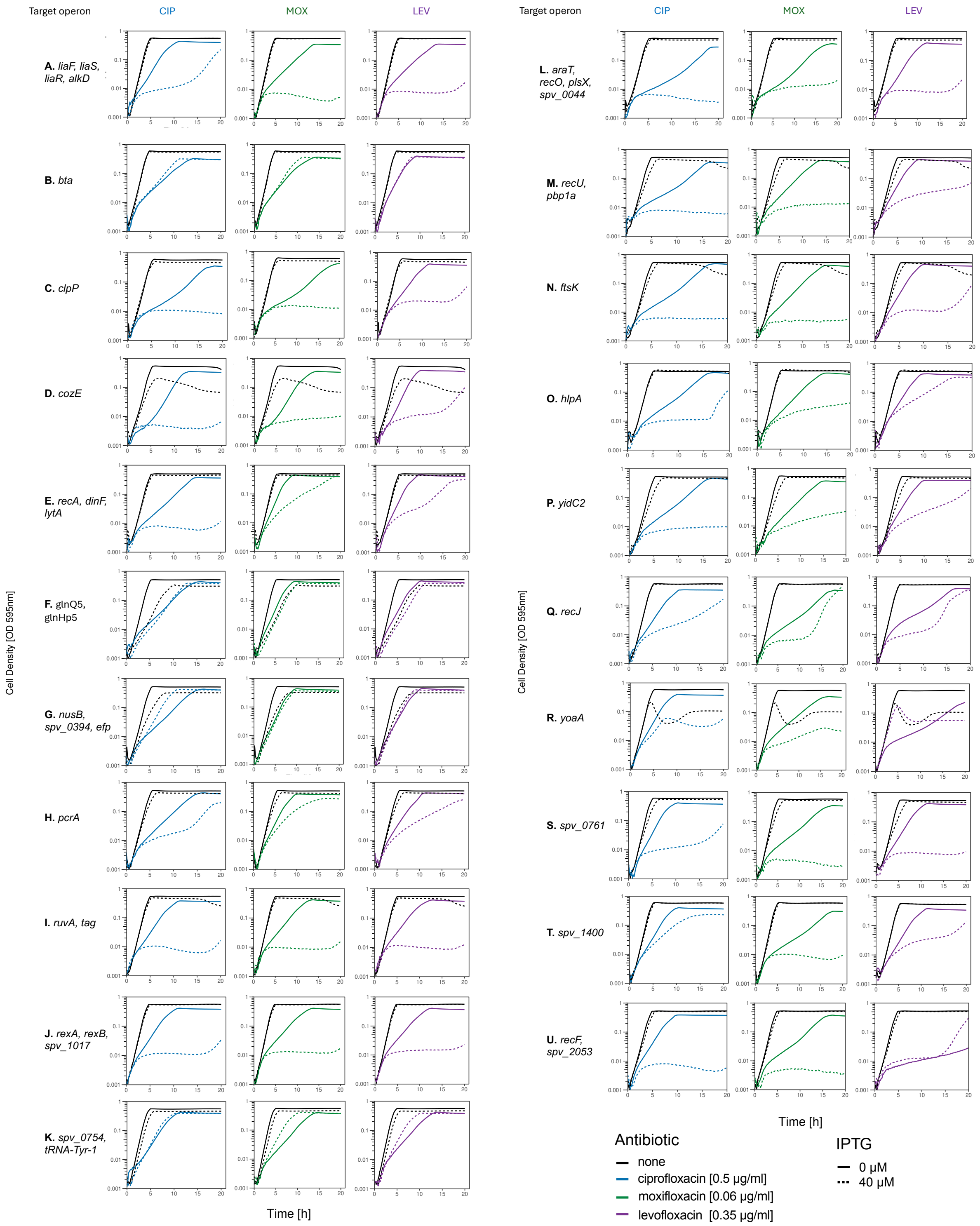

Supplementary Fig 3. Growth profiles of CRISPRi strains treated with fluoroquinolones. (A-U) Individual CRISPRi strains were made for 21 sgRNAs that were some of the top hits from the CRISPRi-seq screens. Strains were treated with CIP, MOX and LEV at sub-lethal concentrations to determine if the growth phenotype of the single mutants could be validated based on the results of the pooled CRISPRi-seq screen. Growth curve data represents the mean ± SEM of three biological replicates (n= 3). Source data are provided as a Source Data file.

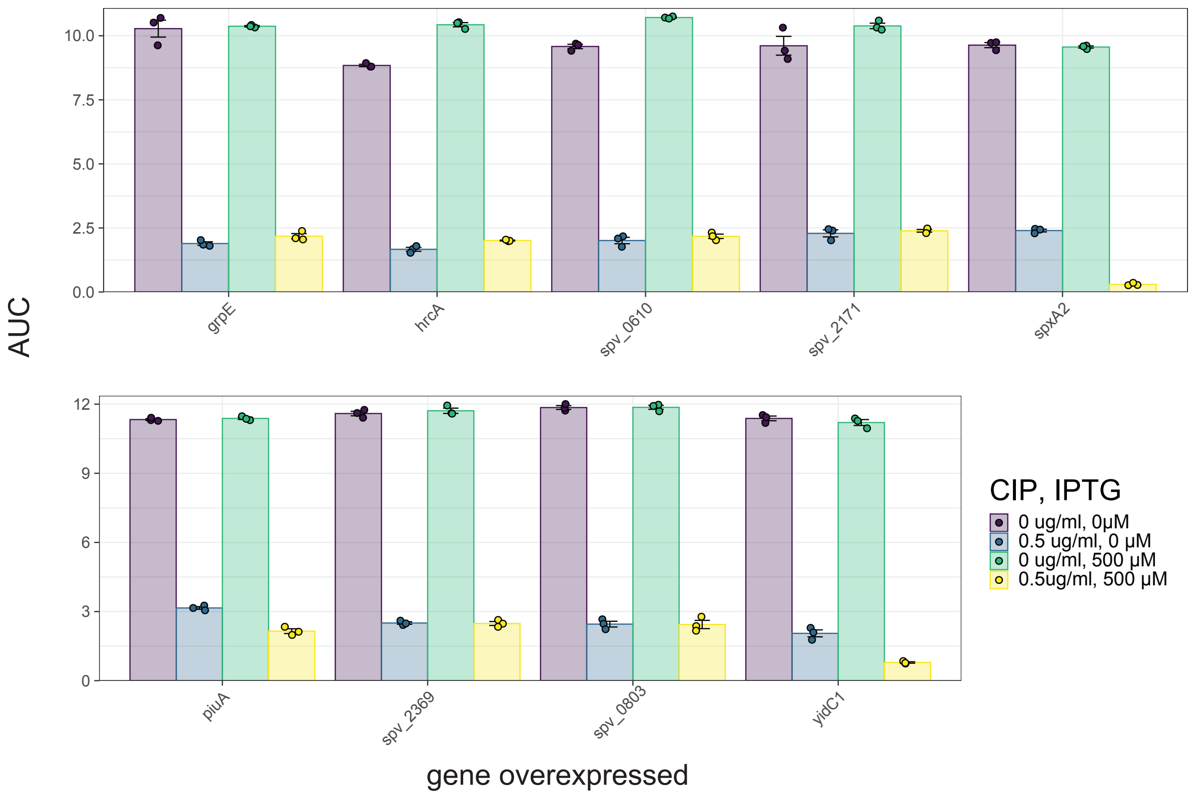

Supplementary Fig 4. Area under the curve (AUC) plots for overexpression strains. Overexpression strains were generated by placing a second copy of the gene at an ectopic locus under the control of p_{lac} and inducing with IPTG. Strains were treated with a sub-lethal concentration of CIP and effect on growth was assessed for 20 hours. Growth data represents the mean ± SEM of three biological replicates (n= 3). Source data are provided as a Source Data file.

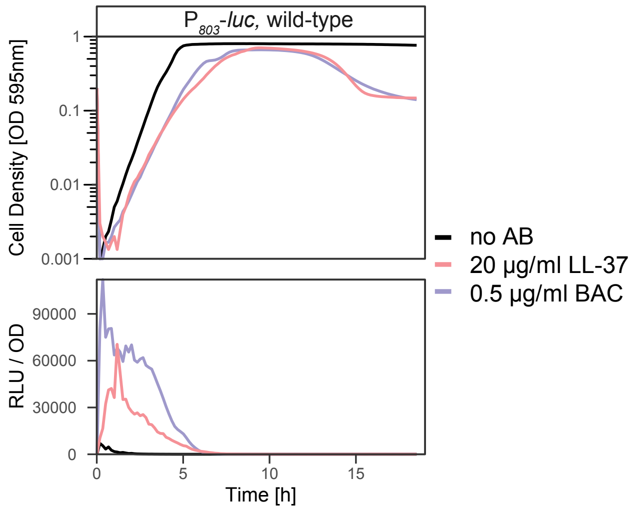

Supplementary Fig 5. LL-37 induced the LiaR regulon. In addition to bacitracin, the antimicrobial peptide LL-37 also induced expression of luciferase from the strain P_{spv_0803}-*luc*, which served as a proxy for LiaR induction. Growth curve data represents the mean ± SEM of three biological replicates (n= 3). Source data are provided as a Source Data file.

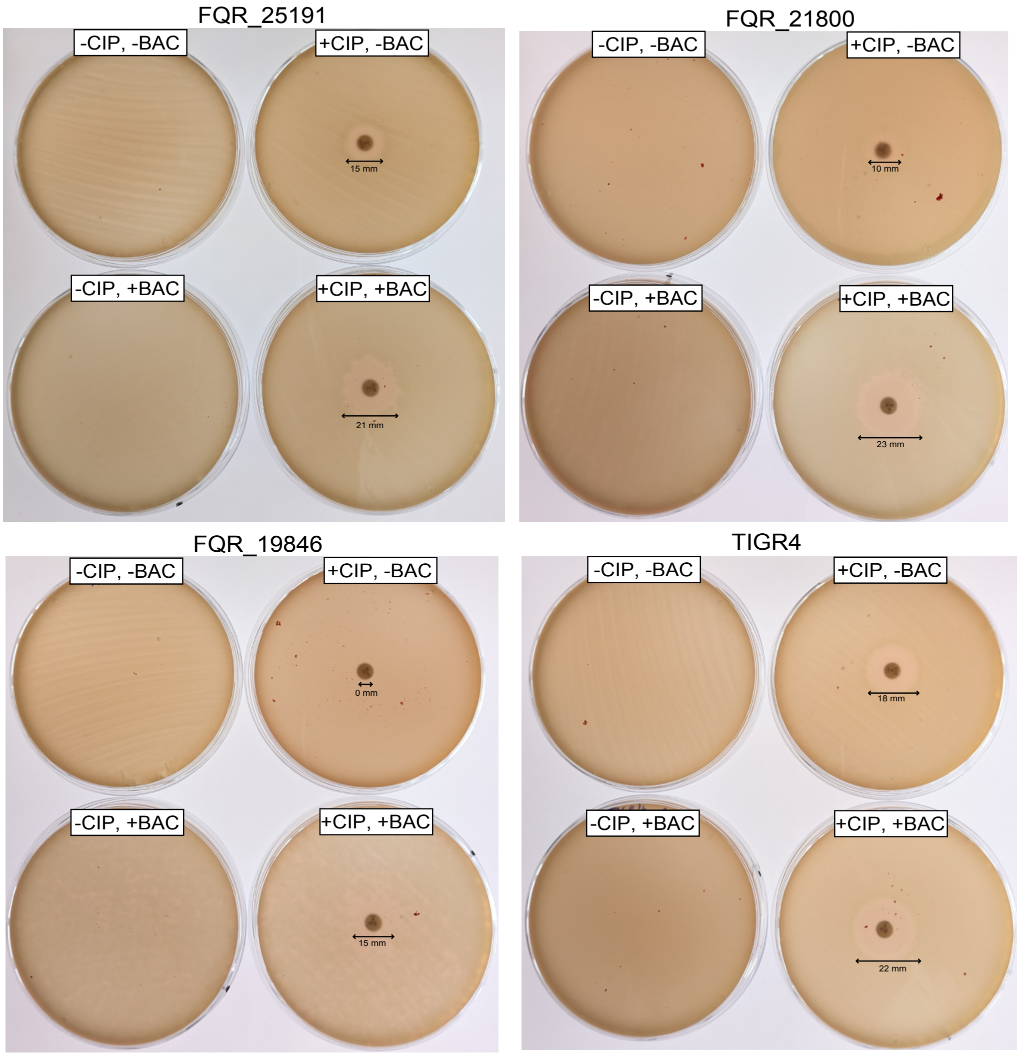

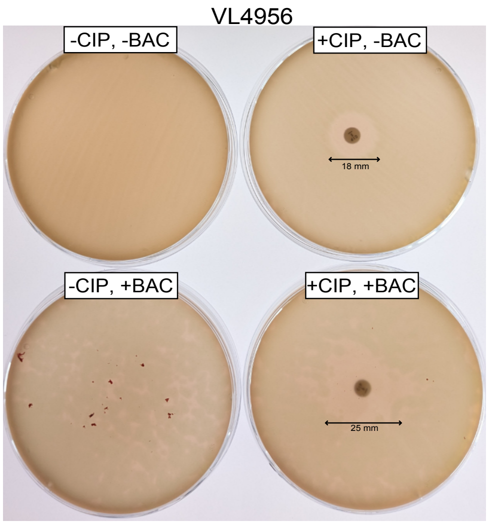

**Strain**	**Isolate description**
FQR_25191	LEV^{R} clinical isolate from sputum, serotype 31
FQR_21800	LEV^{R} clinical isolate from sputum, serotype 19F
FQR_19846	LEV^{R} clinical isolate from trachea, serotype 9V
TIGR4	Lab strain, serotype 4
VL4956	D39V, CIP^{R} *parC* S79F

Supplementary Fig 6. Ciprofloxacin and bacitracin synergize against fluoroquinolone resistant clinical pneumococcal strains. Ciprofloxacin (5 mg/ml) disc diffusion assays show an increased zone-of-inhibition with the addition of 0.5 µg/ml bacitracin (BAC) for several pneumococcal strains of different serotypes and fluoroquinolone resistance profiles.

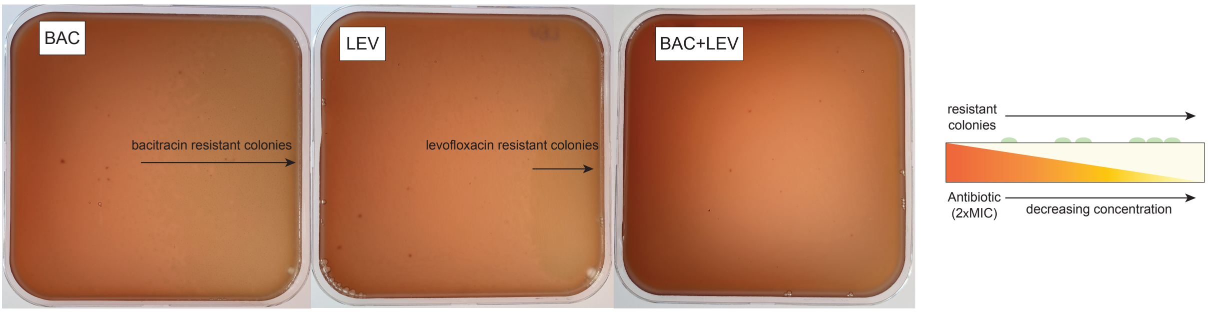

Supplementary Fig 7. No resistant colonies were observed when WT *S. pneumoniae* was exposed to a combination of bacitracin and levofloxacin. Concentration gradient plates show that resistant colonies could be obtained for bacitracin (BAC) and levofloxacin (LEV) individually at 2×MIC concentrations. LEV was used at a concentration of 2 μg/ml and BAC at a concentration of 16 μg/ml. However, no resistant colonies were obtained when bacitracin and levofloxacin were in combination.

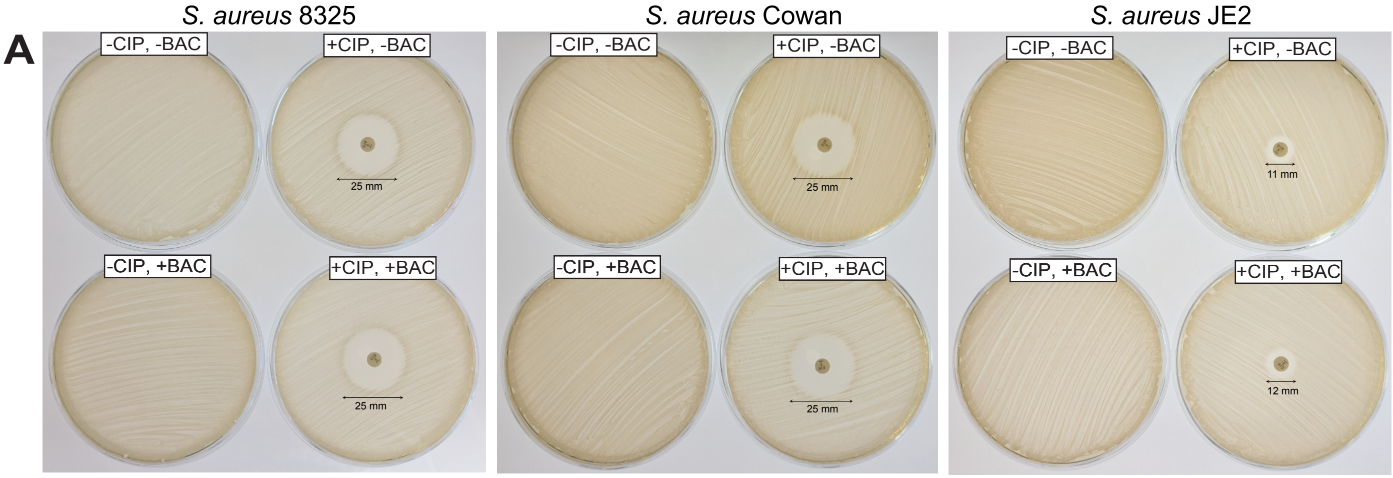

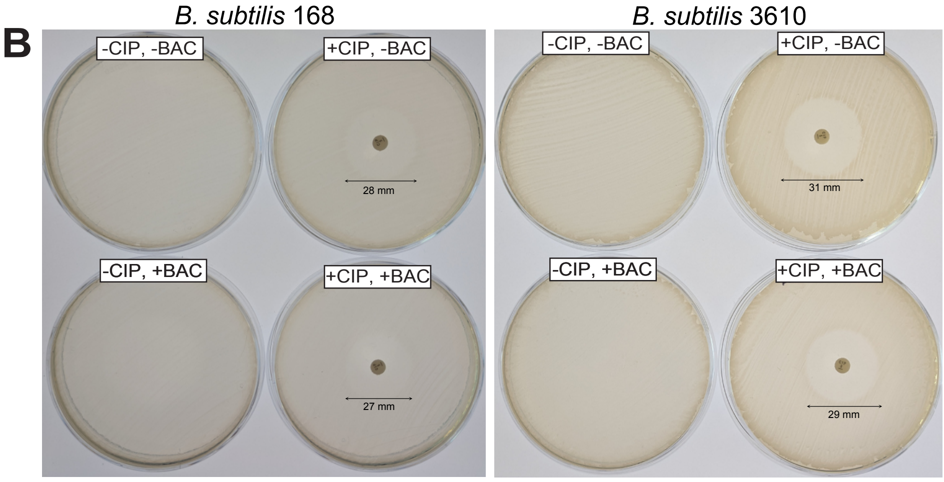

**Strain**	**Isolate description**
S. aureus NCTC8325-4	Lab strain
S. aureus Cowan	Clinical isolate from septic arthritis
S. aureus JE2	Methicillin^{R}, CIP^{R} clinical isolate from skin
B. subtilis 168	Lab strain
B. subtilis NCIB 3610	Wild-type, biofilm-producing strain

Supplementary Fig 8. Ciprofloxacin and bacitracin do not synergize against two other Gram-positive species. Ciprofloxacin (CIP, 5 mg/ml) disc diffusion assays for different strains of **A)** *S. aureus and* **B)** *B. subtilis.* There was no difference in the zone of inhibition between CIP alone and when combined with 0.5 µg/ml bacitracin (BAC) for strains of both species.

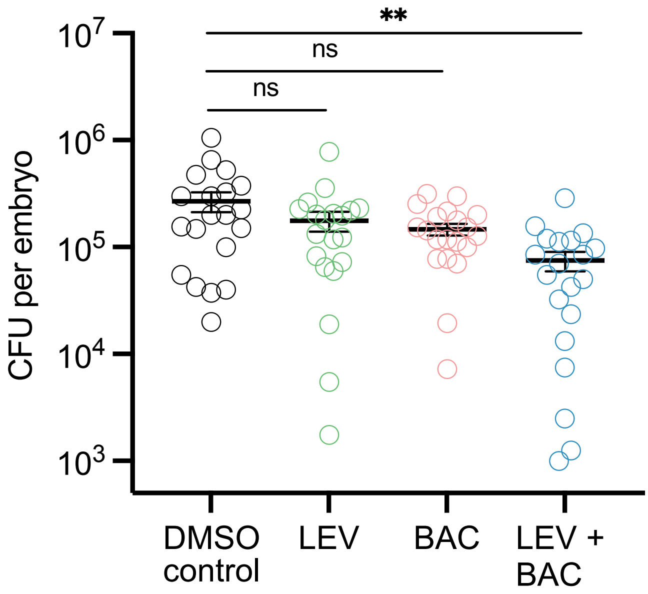

Supplementary Fig 9. Survival of zebrafish directly correlated to a decrease in bacterial CFU burden. Zebrafish embryos were infected with ~400 CFUs of *S. pneumoniae* D39V at 2 days post fertilization and treated at 1 hour post injection with: DMSO vehicle controls, BAC injected into the bloodstream, LEV added into the water, or a combination of both LEV added to the water and BAC injection. Bacterial load (CFU per embryo) was determined at 24 hours post injection. The data represent the mean ± SEM of two biological replicates with 10 embryos per group (n = 20 in total/group); each dot represents a single larva; ns = not significant, **p =0.0022; determined by unpaired t-test.

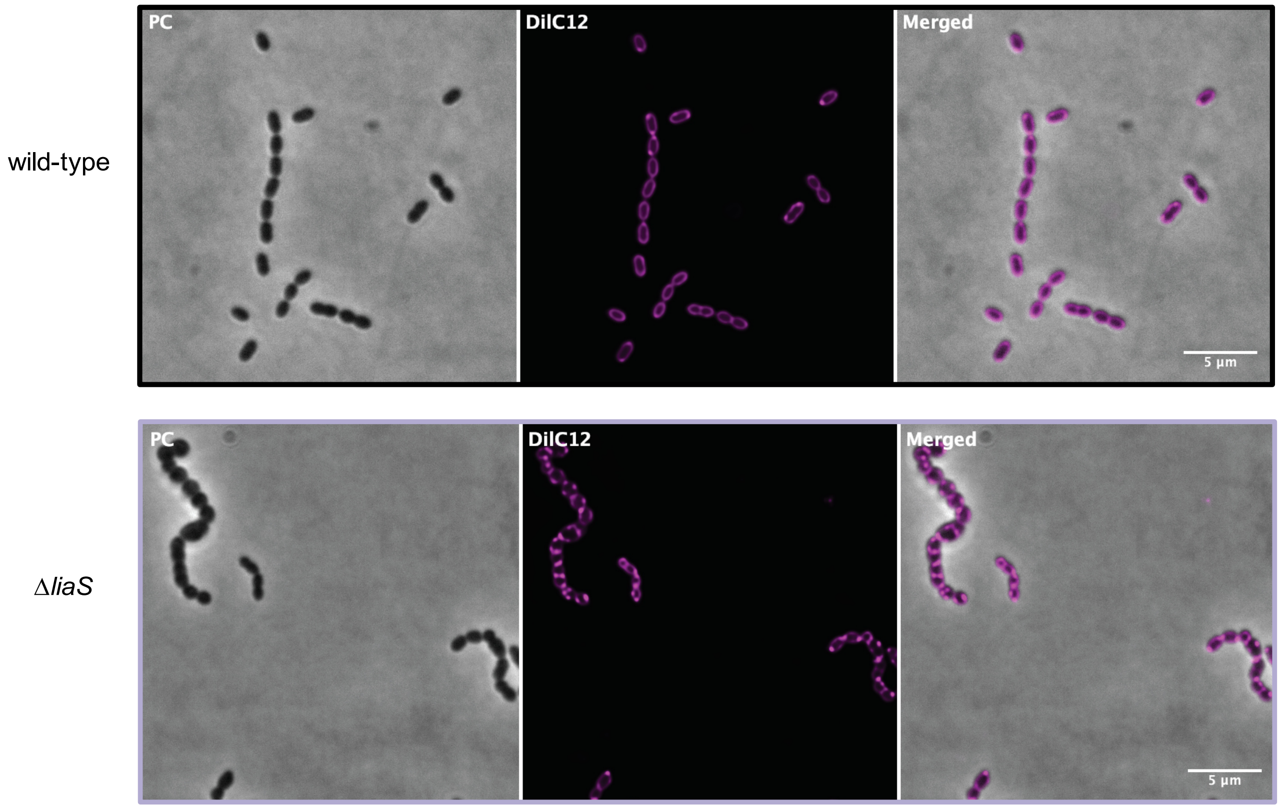

Supplementary Fig 10. The Δ*liaS* mutant displays perturbed cell membrane physiology compared to WT, as visualized by DilC12 staining. Dil-C12 dye incorporates into fluid areas of the cell membrane lipid bilayer, thus allowing visualization of cell membrane integrity. The Δ*liaS* mutant cells displayed irregular cell morphologies with either swollen or shrunken cells. Patches of fluorescent foci could be observed due to the accumulation of Dil-C12 to these highly fluid lipid regions, indicating disruption of the cell membrane.

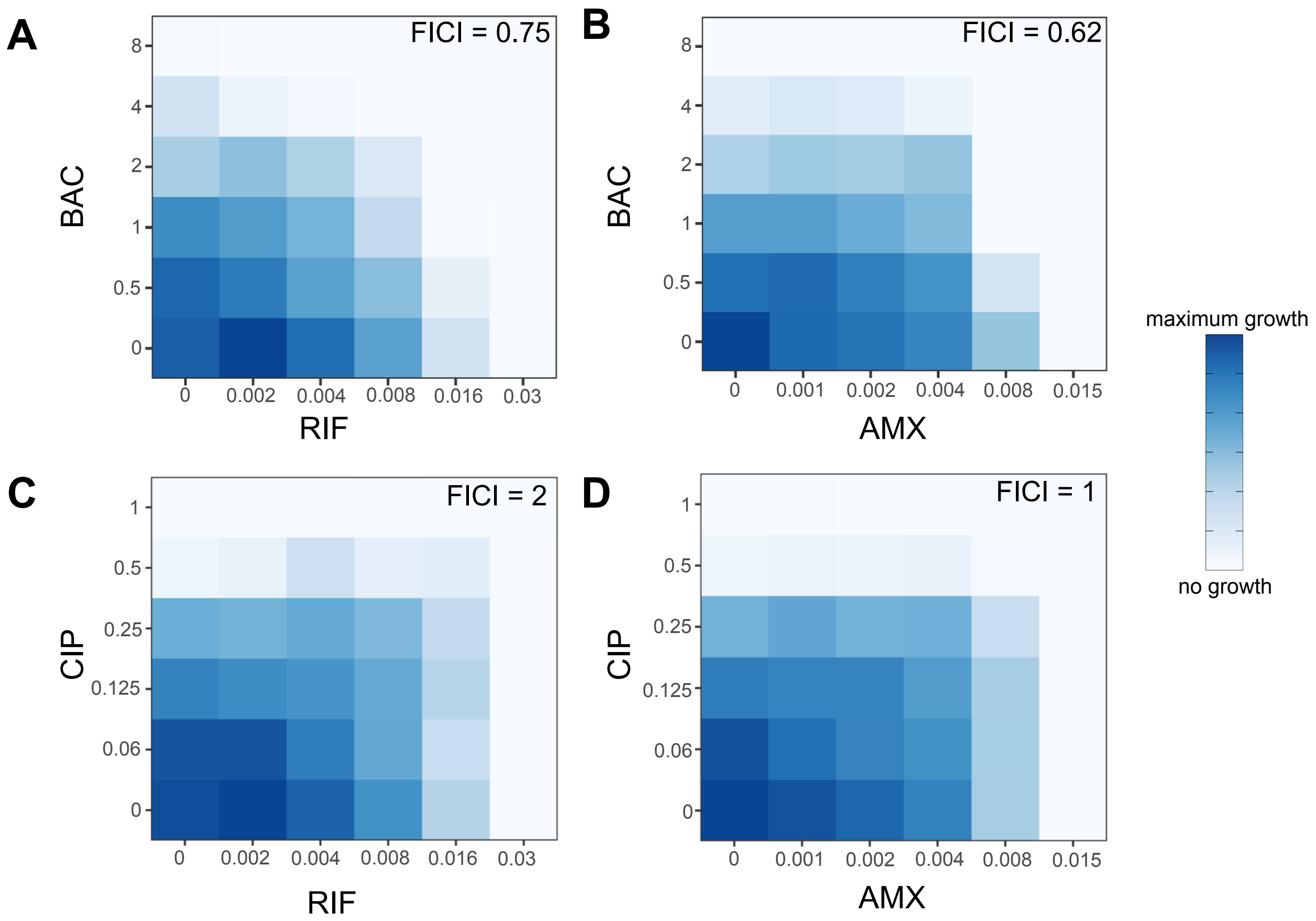

Supplementary Fig 11. Absence of synergy between amoxicillin and rifampicin in combination with ciprofloxacin and bacitracin. Checkerboard assays between **A)** BAC and rifampicin (RIF), **B)** BAC and amoxicillin (AMX), **C)** CIP and RIF, **D)** CIP and AMX indicate these antibiotic combinations do not exhibit a synergistic effect as determined by FIC indexes greater than 0.5 (synergy defined as FICI≤0.5). The combinations are either additive with 0.5<FICI≤1 or indifferent with 1<FICI<4. This suggests that bacitracin and ciprofloxacin do not act as broad-spectrum sensitizing agents.
