# Supplementary material for: Genome-wide antibiotic-CRISPRi profiling identifies LiaR activation as a strategy to resensitize fluoroquinolone-resistant Streptococcus pneumoniae
Source: Nat Commun. 2025 Jul 14;16:6491. doi: 10.1038/s41467-025-61814-x (PMC12260033; doi:10.1038/s41467-025-61814-x)
Supplement: Supplementary file 2 — Description of Additional Supplementary Files [file 41467_2025_61814_MOESM2_ESM.docx]

**Description of Additional Supplementary Files**

Supplementary Data 1: Normalized sgRNA counts from fluoroquinolone CRISPRi-seq screens.

Supplementary Data 2: RNA-seq data generated using DESeq2 indicating genes differentially expressed in an *S. pneumoniae* *∆liaS* mutant compared to wildtype. Statistical significance was defined as |log2FC | > 1, and *P*_adj_ < 0.05 (DESeq2 differential enrichment analysis using a negative binomial generalized linear model with two-tailed P-values adjusted for false discovery rate (FDR)).

Supplementary Data 3: Strain list indicating all strains used in this study.

Supplementary Data 4: Primers used in this study.
